# Cancer-associated retinopathy after surgery for breast cancer: a case report and review of the literature

**DOI:** 10.1186/s40792-018-0418-3

**Published:** 2018-01-23

**Authors:** Mirei Kamei, Yutaka Fujitomi, Yoshiyuki Kondo, Toru Adachi, Kohei Shibata, Yohei Takumi, Miyuki Abe, Kenji Sugio

**Affiliations:** 1 Department of Surgery, Tsurumi Hospital, 4333 Tsurumi, Beppu, Oita 874-8585 Japan; 2 Department of Pathology, Tsurumi Hospital, 4333 Tsurumi, Beppu, Oita 874-8585 Japan; 3 Department of Ophthalmology, Faculty of Medicine, Oita University, 1-1 Idaigaoka, Hasama, Yufu, Oita 879-5503 Japan; 4 Department of Thoracic and Breast Surgery, Faculty of Medicine, Oita University, 1-1 Idaigaoka, Hasama, Yufu, Oita 879-5503 Japan

**Keywords:** Cancer-associated retinopathy, Invasive carcinoma with neuroendocrine features, Steroid pulse therapy

## Abstract

We herein report a 50-year-old Japanese woman with breast cancer who complained of blurred vision and central scotoma in her left eye on the 12th day after surgery. Subsequently, the sudden-onset binocular visual disorder progressed, and she was diagnosed with cancer-associated retinopathy (CAR) based on the clinical findings. Although her visual acuity temporarily improved following the start of adjuvant chemotherapy, reductions in her visual acuity progressed once again. After two courses of steroid pulse therapy initiated from the 59th day following the onset of CAR, although her visual field was still constricted, her binocular visual acuity improved from finger movement to 0.8 2 months later. The shorter the period from onset to treatment, the better the prognosis of the visual function. However, a diagnosis is often delayed because the incidence of this disease is very rare. Therefore, it is important to suspect CAR whenever a sudden visual disorder develops in cancer patients. Furthermore, treatment is believed to be effective even if steroid therapy is started up to 2 months from onset.

## Background

Cancer-associated retinopathy (CAR) is an extremely rare paraneoplastic syndrome that causes subacute retinal vision disorders. CAR caused by a common antigen between tumor cells and the retinal vision system targeted against retinal antigens. CAR suddenly and progressively manifests symptoms similar to retinitis pigmentosa, such as a reduction in the visual acuity and constriction of the visual field. CAR has been reported as leading to the detection of malignant tumors in many cases, particularly small-cell lung cancer, although various tumors (including benign ones) can occur with CAR.

We herein report a case of CAR that developed 12 days after breast cancer surgery, along with a literature review.

## Case presentation

**Fig. 1:**
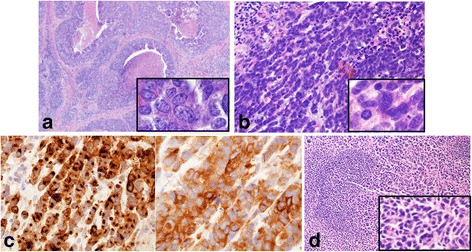
Microscopic findings of resected specimen. **a** The tumor cells of invasive ductal carcinoma show round to oval hyperchromatic nuclei with prominent nucleoli. **b** The tumor cells of neuroendocrine features have small scant cytoplasm and finely granular hyperchromatic nucleus without nucleoli. **c** The tumor cells are highly immunopositive for both chromogranin A (left) and synaptophysin (right). **d** The metastasis of axillary lymph node is caused component of neuroendocrine features

A 50-year-old Japanese woman was referred to our hospital with calcifications in the right breast discovered on mammography at a medical checkup. She had no family history of abnormalities or medical history of particular note. Upon a detailed examination, she was diagnosed with breast cancer. Tumor markers (CEA, CA15-3, BCA225) were all within normal limits. The computed tomography scan showed one axillary lymphadenopathy and no distant metastasis. Radical mastectomy and axillary dissection were performed. The pathological diagnosis was invasive carcinoma with neuroendocrine features, with axillary lymph node metastasis caused by component of neuroendocrine features (Fig. 1a–d).

There was a high level of lymphatic invasion, and 2/15 lymph nodes tested positive for malignancy with a maximum tumor diameter of 2.7 cm. The component of neuroendocrine features was nuclear grade 2, estrogen receptor (ER)-positive (< 10%), progesterone receptor (PgR)-negative (< 1%), and human epidermal growth factor 2 (HER2)-negative, while the invasive ductal carcinoma was nuclear grade 3, ER-positive (< 50%), PgR-positive (< 50%), HER2 equivocal (2+), and unamplified on fluorescence in situ hybridization (FISH). The cells of both tumors had a high Ki-67 proliferation rate (over 99% of total tumor cells).

**Fig. 2:**
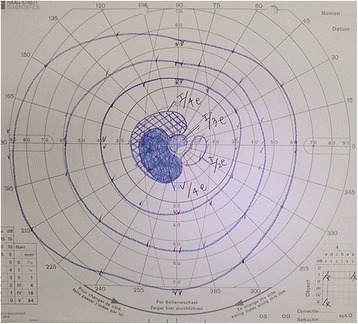
The Goldman perimeter indicated paracentral visual field defects in her left eye

At 12 days after surgery, she visited the Department of Ophthalmology due to the sudden onset of blurred vision in her left eye. Although photopia, central scotoma, and a reduction in her visual acuity were observed, a fundoscopy examination revealed almost no abnormal retinal findings. The pupils were almost normal with no relative afferent pupillary defect. The Goldman perimeter indicated paracentral visual field defects in her left eye (Fig. 2). After 1 week, ocular symptoms also developed in her right eye along with an increase in the intraocular pressure of her left eye, so laser therapy was performed. Due to constriction of her binocular visual field, a reduction in her visual acuity, and the progression of photophobia, brain metastasis was suspected. However, brain magnetic resonance imaging (MRI) showed no metastasis or any intracranial lesions.

**Fig. 3:**
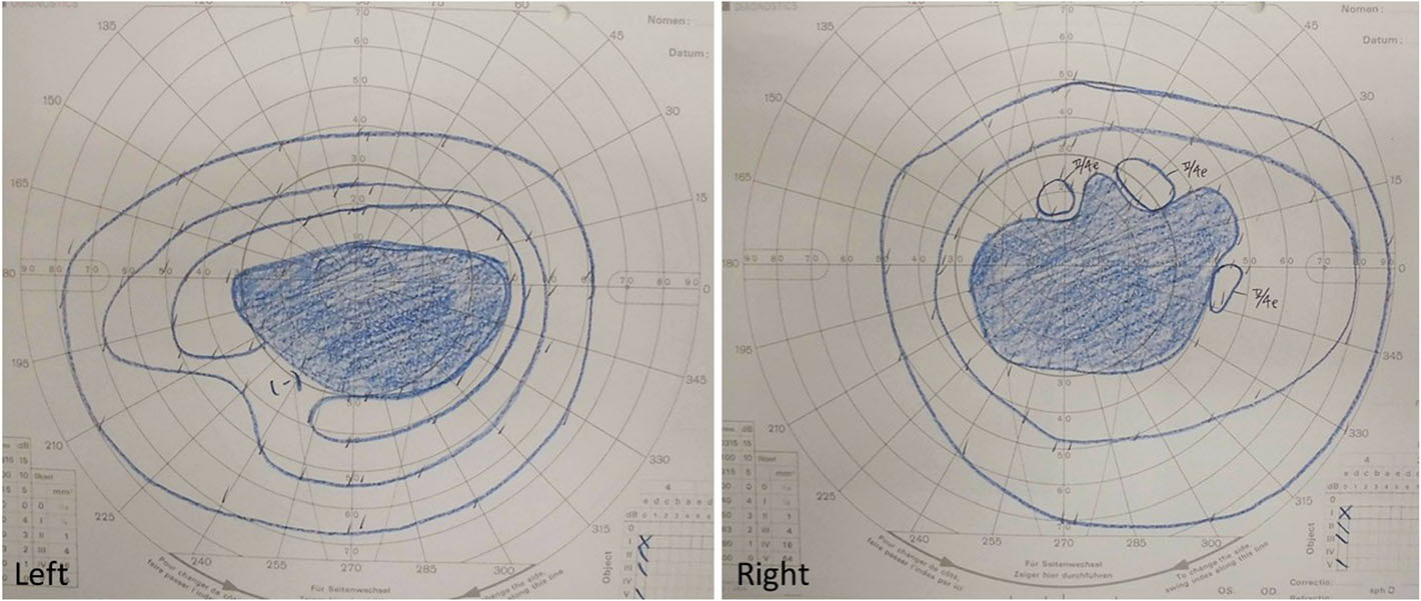
Visual field examination demonstrates a large central scotoma in both eyes

During the 3 weeks following the onset of her vision disorder, her vision acuity and photophobia worsened. Although CAR was suspected, anti-recoverin antibody was not detected. However, a diagnosis of CAR was ultimately made due to her ophthalmological findings and clinical symptoms. Although the final diagnosis of the tumor was invasive carcinoma with neuroendocrine feature, the initial diagnosis was small cell carcinoma of the breast. So, we selected cisplatin plus etoposide based regimen for this patient on the 29th postoperative day. Although photophobia remained on the seventh day following the start of chemotherapy, her left visual acuity temporarily improved. However, after 2 weeks, reductions in her visual acuity began to progress once again. A visual field examination revealed a large central scotoma, and she lost light perception in both eyes (Fig. 3). On the 56th postoperative day, follow-up whole-body computed tomography revealed local recurrence in the axilla. Therefore, chemotherapy was suspended, and radiotherapy and tamoxifen therapy was initiated. From the 59th day following the onset of CAR, She was treated with steroid pulse therapy (methylprednisolone 1000 mg/day, 3 days) in 2-week intervals for two courses. On the sixth day following the first course, her left visual acuity improved from finger movement to 0.06. Although the central scotoma and photophobia did not improve, her binocular visual acuity recovered to 0.2 on the seventh day after the second course, followed by a recovery to 0.8 on the 35th day after the second course.

**Fig. 4:**
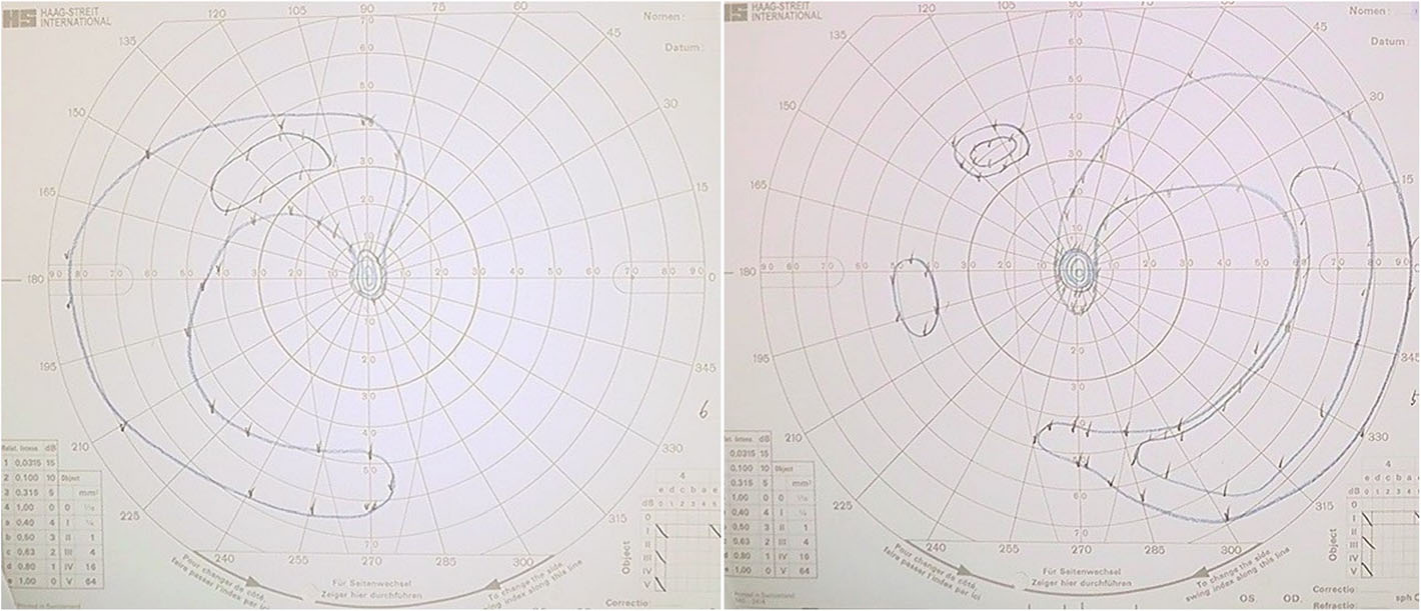
Although visual field revealed concentric contraction in both eyes, central visual field of the both eyes are maintained

Twelve months following the onset of CAR, the paracentral and peripheral scotoma remain, and she is now forced to walk with a cane and cannot drive a car (Fig. 4). However, she has maintained binocular vision of 1.2. Since the adjuvant chemotherapy was necessary for invasive breast carcinoma based on the final pathological diagnosis after radiotherapy, she received 4 cycles of chemotherapy with epirubicin and cyclophosphamide followed by 4 cycles of chemotherapy with docetaxel from the 92th postoperative day. She has subsequently been maintained on endocrine therapy. The axillary metastasis has disappeared, and there is no evidence of any other recurrence at 16 months after surgery.

### Discussion

The concept of CAR was first reported by Sawyer et al. in 1976 in their paper on the association of idiopathic vision disorders with cancer patients [1]. In 1991, Polans et al. reported that the autoantibody associated with CAR was recoverin [2]. Since then, multiple autoantibodies related to CAR have been identified, and a number of teams have been researching other as-yet-unknown antibodies. Small-cell lung cancer is the most commonly reported primary lesion, followed by other solid cancers, such as gynecologic cancer, gastrointestinal cancer, and breast cancer; hemoblastosis, such as malignant lymphoma and leukemia; sarcoma; and thymoma. To our knowledge, only four case reports exist on breast cancer, including the present case, with this study alone having reported invasive carcinoma with neuroendocrine features.

Table 1 [3–5] summarizes the cases of CAR associated with breast cancer reported to date. The average age was 56.4 (50–62) years. Adamus et al. reported that the onset of CAR in breast cancer was more common among women ≥ 50 of age, around the time of menopause, which is consistent with this study [6].

**Table 1:** Case reports of cancer associated retinopathy with breast cancer

Author	Age	Symptom	Diagnosis of BC	Stage of BC	Chemotherapy	Treatment of CAR	Antibody	From CAR onset to treatment	Course	Pathology
Holz FG(1997) [3]	53	Blurred vision/reduce of dark adaptation paracentral scotoma	5 years 1 month before	T2N0M0	Done (epirubicin/treosulfan)	Steroid therapy methotrexate	Recoverin: -	No record	No remission	No record
	62	Photopsia photosensitivity blurred vision	6 years before	T1bN0M0		Steroid therapy methotrexate	No performed	4 months	No remission	No record
Ejma M(2008) [4]	54	Decreased vision in the dark photopsia photosensitivity	8 months before	T4dN0M+	Done (FAC)	None	Recoverin: - α-enolase: + GCAPs: - retinal arrestin: -	–	No remission (died)	No record
Anastasakis(2011) [5]	62	Shimmering Photopsia myodesopsia	2 months after	TxNxM+(PUL,PAN)	Done (no record)	Chemotherapy	No performed	2 months	Normalized	Lobular ca.
present case	50	Decreased vision photopsia photosensitivity defect of visual field	30 days before	T2N1M0	Done (CDDP+VP16,EC,DTX)	Steroid therapy	Recoverin: -	59 days	Improved	Invasive ca. with neuroendocrine features

*BC* Breast cancer, *FAC* Fluorouracil and doxorubicin and cyclophosphamide, *CDDP* cisplatin, *VP-16* Etoposide, *EC* Epirubicin and cyclophosphamide, *DTX* Docetaxel, *PUL* Pulmonary, *PAN* pancreas

Four cases developed CAR after the diagnosis of breast cancer, among which one case was diagnosed with breast cancer with metastasis based on a detailed examination for a vision disorder. Among the case reports including other tumors to date, CAR has led to the discovery of primary tumors in many cases. However, Adamus et al. reported that the anti-retinal cell line antibodies in many breast cancer patients are already expressed before the onset of breast cancer, causing vision disorders 2 or 3 years after the cancer diagnosis, whereas CAR precedes the diagnosis of cancer in only 5% of cases [6]. One case involved breast cancer with distant metastasis. In that case, which was considered to be T2 stage, while the disorders were caused 30 days after the diagnosis of cancer, it is presumed that several years actually passed from the onset of cancer. Although stages are not mentioned in some of the previous reports, many cases involve advanced cancers, indicating that CAR only incidentally led to its detection. Furthermore, CAR itself is rare and has a low degree of recognition. Unless suspected, appropriate examinations and treatments may not be performed.

Symptoms of CAR often begin with photophobia, photosensitivity, and myodesopsia. The initial symptoms in the present case were central scotoma, photopsia, and photophobia of the left eye, with similar symptoms appearing in the right eye after 1 week; we subsequently observed exacerbation of the visual disorder weekly. Bleeding and vasculitis were not noted on fundoscopy, which revealed only lightly clouded spots scattered in the retina. Previous reports cited three cases involving both eyes, however, the occurrence and progression of which were not necessarily simultaneous and were heterogenous.

CAR is diagnosed based on the clinical symptoms and detection of anti-retinal antibodies in the serum. In previous reports, examinations were carried out in three cases, but anti-recoverin antibody was not detected in all cases, and only one case was actually found to be positive for anti-enolase antibody. Adamus reported that the possibility of anti-enolase antibody positivity was highest, while that of anti-recoverin antibody positivity was lowest in breast cancer [6]. Therefore, had the presence of anti-enolase antibody been examined in the present case, the results might have been positive. We believe that anti-retinal antibody positivity is not necessary in order to make a diagnosis of CAR at this stage.

Various treatments are available for CAR, including steroid pulse therapy, plasmapheresis, immunoglobulin, intravitreal injection of steroids, and chemotherapy for the primary disease. Although intravenous steroid pulse therapy was carried out in the previous three cases, improvement of symptoms was only observed in this study. From the 59th day following the onset of CAR, intravenous steroid pulse therapy was carried out in 2-week intervals for two courses. Her left visual acuity improved, and she was able to read numbers from the fourth day after starting the course. However, even if the visual dysfunction improves transiently, it often recurs. There was no recurrence even 14 months after the onset of CAR in this case, though. While the early diagnosis and early treatment of CAR leads to improvement in the visual function, there have been reports of an improved visual dysfunction even when started after 9 months [7]. However, only one previous case recovered completely [5], so making a perfect recovery seems difficult. In the present case, despite starting the treatment 2 months after the onset of CAR, it was still effective. Considering the balance needed between treatment for CAR and that for the original disease, we should at least consider intravenous steroid pulse therapy to improve patients’ quality of life.

The proliferation of anti-recoverin antibody-positive cancer has been slow, with a good prognosis, as recoverin itself works as a cancer antigen and induces T cell cytotoxic activity [8, 9]. However, according to the previous literature, many cases are already advanced at the time of discovery, indicating the need for further investigation. It has also been reported that the relapse of CAR and tumor recurrence is linked, making it extremely important to monitor the re-exacerbation of CAR going forward.

## Conclusion

We experienced a case of CAR 12 days after breast cancer surgery. CAR should be suspected when sudden vision disorders occur in patients undergoing cancer treatment, and treatment for CAR should be performed promptly considering the condition of the original tumor if the diagnosis is confirmed.

### Consent

Written informed consent was obtained from the patient for publication of this case report and any accompanying images. A copy of the written consent is available for review by the Editor-in-Chief of this journal.

## Abbreviations

BCA225: Breast carcinoma-associated antigen225
CA15-3: Cancer antigen15-3
CEA: Carcinoembryonic antigen
